# Low Voltage Graphene-Based Amplitude Modulator for High Efficiency Terahertz Modulation

**DOI:** 10.3390/nano10030585

**Published:** 2020-03-23

**Authors:** Qianying Zheng, Liangping Xia, Linlong Tang, Chunlei Du, Hongliang Cui

**Affiliations:** 1 Chongqing Institute of Green and Intelligent Technology, Chinese Academy of Sciences, Chongqing 400714, China; zhengqianying@cigit.ac.cn (Q.Z.); tll@cigit.ac.cn (L.T.); hcui@cigit.ac.cn (H.C.); 2 University of Chinese Academy of Sciences, Beijing 100049, China; 3 Key Laboratory of Micro Nano Optoelectronic Devices and Intelligent Perception Systems, Yangtze Normal University, Chongqing 408100, China

**Keywords:** terahertz, amplitude modulation, transmittance, single-layer graphene, metamaterial, solid electrolyte, field-effect transistor

## Abstract

In this paper, a high-efficiency terahertz amplitude modulation device based on a field-effect transistor has been proposed. The polarization insensitive modulator is designed to achieve a maximum experimental modulation depth of about 53% within 5 V of gate voltages using monolayer graphene. Moreover, the manufacturing processes are inexpensive. Two methods are adopted to improve modulation performance. For one thing, the metal metamaterial designed can effectively enhance the electromagnetic field near single-layer graphene and therefore greatly promote the graphene’s modulation ability in terahertz. For another, polyethylene oxide-based electrolytes (PEO:LiClO_4_) acts as a high-capacity donor, which makes it possible to dope single-layer graphene at a relatively low voltage.

## 1. Introduction

The electromagnetic spectrum from 0.1 to 10 terahertz (THz) is called the THz region. As they have superior intrinsic properties, terahertz waves can interact with various materials and have a wide range of applications, such as penetrating imaging [1], high-speed wireless communication [2], and medical sensing [3]. Among these, the THz modulator is an essential part of all sophisticated THz application systems [4,5,6].

Similar to the active modulators of optical and microwave regimes as reported, the established ones in THz are also in the demand for high efficiency, fast modulation speed, and low power consumption [7]. Graphene is a kind of zero-band-gap two-dimensional material that is well known for its remarkable carrier mobility (~15,000 cm^2^V^−1^s^−1^) without a high incident excitation source at room temperature [8]. Therefore, it is a promising candidate for THz active modulators [9].

Although some progress has been made on electrically tunable graphene modulators in THz, the performance of such modulators still needs enhancing. One of the basic focuses is on increasing the modulation depth (*MD*) while lowering the drawing-voltage.

The monatomic layer thickness of graphene (~0.36 nm) is disadvantageous for strong interactions with electromagnetic waves passing through, which will inevitably lead to a small *MD* [10,11]. Therefore, artificial metamaterial, one of the effective approaches, has been brought forward to enhance the light-matter interaction of graphene [12,13,14,15]. According to the previous study, the electromagnetic fields surrounding graphene could be extremely magnified, over a hundred times, by integrating graphene with metamaterial [16].

In addition, another deficiency is that the drawing-voltage of THz graphene modulators is generally as high as several tens of volts [13,17]. Given all these dynamic devices are controlled by manipulating the carrier concentration of graphene, enhancing doping efficiency will be a crucial point to realize deep tunable elements with low voltage. Compared with conventional materials adopted, the solid electrolyte is one of the attractive options for high-capacity donors applied in THz. For instance, the capacitance of solid electrolyte (10 μFcm^−2^) is ≈800 times higher than that of the 300 nm SiO_2_ plate capacitor [18,19,20]. Moreover, unlike high-k dielectrics (1 to 2 μFcm^−2^) [21,22,23,24,25,26], solid electrolytes would not have problems due to the strict constraints of the growth environment or the processing conditions [27]. Aside from this, it was reported that the solid electrolyte also had superior advantages including transparency, good mechanical flexibility, fatigue stability, electrochemical, and thermal stability [28,29,30].

In this work, we propose an effective THz configuration comprised of metamaterials, solid electrolyte, and graphene to realize dynamical modulation of device transmittance. By using the solid electrolyte top gate, we can obtain a highly doped graphene membrane with activity tunable carrier densities via small gate voltage. Moreover, it has been experimentally verified that a maximum *MD* of 53% is achieved within 5 V of gate voltages with this device. Besides, the fabrication of this device demands inexpensive equipment. This architecture provides a feasible approach for optimizing THz active modulation performance with single-layer graphene. Additionally, because the flower-shaped structure in this work is considered a kind of customized metamaterial, it could be replaced corresponding to a specific application in the future.

## 2. Methods and Results

**Figure 1:**
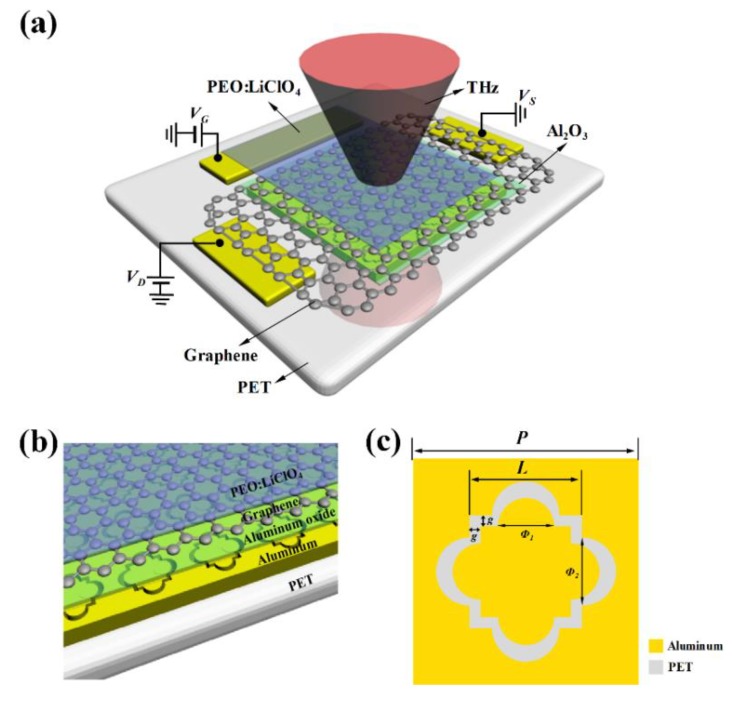
The global and local schematic diagrams of the proposed modulator. (**a**) Schematic diagram of the whole terahertz (THz) modulation device. Only one gate electrode is demonstrated here while the other, at the symmetrical side of the functional section, is removed for explicit annotation. There are some abbreviations: PET (polyethylene terephthalate), PEO (polyethylene oxide), *V_G_* (gate voltage), *V_D_* (drain voltage); *V_S_* (source voltage). (**b**) The detailed structure of the functional section. There are five layers from the bottom to the top; (**c**) Schematic of the unit cell of the aluminum metamaterial marked with dimensional details. *P* = 200 μm, *L* = 100 μm, *g* = 10 μm, $\Phi_{1}$ = 50 μm, $\;\Phi_{2}$ = 60 μm. The thickness of polyethylene terephthalate, aluminum, and alumina layers are 35 μm, 500 nm, and 50 nm respectively.

The schematic of the THz amplitude modulator is shown in Figure 1a. The device in the form of a field-effect transistor (FET) could be divided into two parts: the central functional section and the surrounding electrodes section, whose positions are demonstrated through Figure 1a.

The central functional section of this modulator is composed of five layers from the bottom to the top, as demonstrated in Figure 1b. The transparent substrate supporting the other components is polyethylene terephthalate (PET), which is an ideal material due to its low loss in the terahertz band and outstanding corrosion resistance [31]. Then, the aluminum metamaterial in Figure 1c was placed on the substrate. The metallic array is insensitive to polarization because each unit could coincide with the original pattern every ninety degrees rotation. It could stimulate specific resonance modes to enhance the local electric field. Then, the metallic surface was coated with an alumina spacer to prevent the single-layer graphene (SLG) from directly touching the metal layer. This step helps to prevent the graphene strip from being highly doped by free electrons, which makes the Dirac point not shift far from the gate voltage 0 V and decreases the modulation difficulty of the solid electrolyte. Afterward, the SLG was transferred onto the dielectric gap, not only to connect the source-drain electrodes, but also to compose the dynamic part of the device. On top of the graphene layer, the solid electrolyte of polymer electrolyte (PE), consisting of poly (ethylene oxide) (PEO) and lithium perchlorate (LiClO_4_), are employed to cover the modulation region and gate electrodes. Voltages applied on the top gate can electrolyze solid electrolyte into Li^+^ and ClO_4_^−^ ions [32], and then these free ions would selectively accumulate and enter the graphene channel. As a result, electric conductivity, namely the original charge density (~10^12^ cm^−2^) [33] and Fermi energy (*E_f_*) in the monolayer graphene, can be significantly changed using drawing-voltages [20].

**Figure 2:**
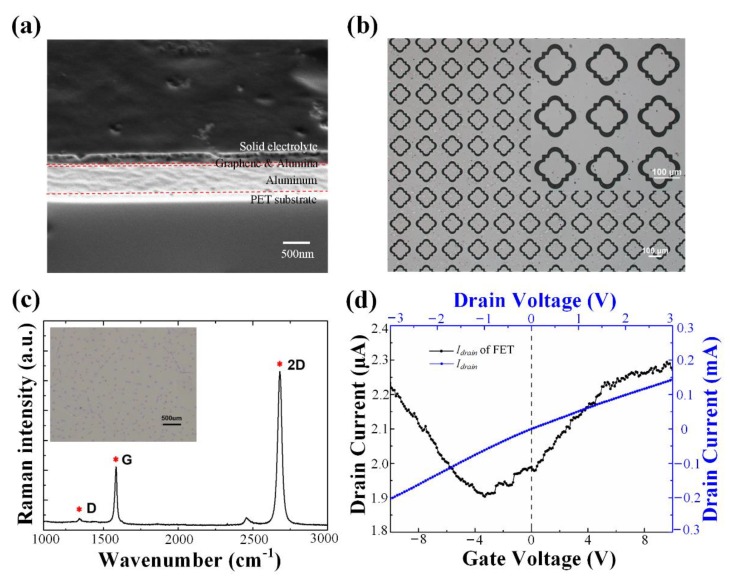
The experimental results of the designed modulator. (**a**) The cross-section of the functional region of a modulator. The observed window is dug and observed by a focus ion beam (FIB, Carl Zeiss, Oberkochen, Germany), while the polyethylene terephthalate (PET) substrate is unpenetrated. The boundaries of each layer can be recognized by color and texture; (**b**) Micrographs of the flower-shaped structure in an aluminum film with different magnification; (**c**) Raman spectrum of a graphene sheet on SiO_2_. Inset: optical microscopic photo of this transferred flake; (**d**) Transfer characteristic of the device as a function of gate voltages (black dots and line), and conductive current of graphene stripe as a function of source-drain voltages (blue dots and line).

Structures of the fabricated functional region are shown in Figure 2a, which implies the fabrication steps of this device from bottom to up intuitively.

Firstly, the metallic structure and electrodes are fabricated through wet etching. Specifically, PET with 500 nm thick aluminum, the raw material bought directly (Changyu Materials Ltd., Tai’an, China), was spin-coated with 1 μm thick S1805 photoresist (Shipley Co., Marlborough, MA, USA) and exposed to ultraviolet light under a mask (ABM Inc., San Jose, CA, USA), to pattern the photoresist with flower-shaped structures. Then, the membrane with developed photoresist was immersed into the corrosive liquid (Shipley Co., USA) for 80 s to remove the unwanted part of the aluminum layer. The etchant mentioned was comprised of phosphoric acid, H_2_O, acetic acid, and nitric acid (Adamas Reagent Ltd., Shanghai, China), with a volume ratio of 16:2:1:1. By carefully selecting the immersion time, the depth and width of etched trenches could be controlled to form accurate and uniform large-area structures, as presented in Figure 2b. To be compatible with the waist radius of our terahertz time-domain spectroscopy (THz-TDS) system (Advanced Photonix Inc., Camarillo, CA, USA) whose diameter is 0.8 cm, the functional region was set as 1.5 cm × 1.5 cm, and the distance between the metamaterial array and electrodes was 1.5 mm. Besides that, gate electrodes are set as 1.5 cm × 0.7 cm and distributed symmetrically on both sides of the central section.

Secondly, an alumina spacer (Zhong Nuo Xin Cai Ltd., Beijing, China) with a thickness of 50 nm was deposited on metamaterial film by electron beam evaporation (Sky Technology Development Co., Shenyang, China). In the practical process, a hard mask was used to shield the electrodes.

Thirdly, a commercially chemical vapor deposition (CVD)-grown graphene strip (Xianfeng Nano Inc., Nanjing, China) was carefully transferred via the poly(methyl methacrylate) (PMMA, Adamas Reagent Ltd., Shanghai, China)-assisted wet-transfer process onto the dielectric layer to connect the source with drain electrodes [34]. Given that the original graphene was grown on both sides of the copper foil, further processing is needed to get rid of the unwanted side. To be specific, one side of the copper foil was coated with 1 μm thick PMMA to support and protect the target graphene, while the other side was etched with oxygen ion (Beijing Ailan Technology Ltd., Beijing, China) to remove graphene. Then, the film was snipped to a moderate size (1.8 cm × 1.3 cm in this work), and the copper membrane was dissolved by etchant comprising of hydrogen peroxide, hydrochloric acid, (Adamas Reagent Ltd., Shanghai, China) and deionized water, with a volume ratio of 1:2:20. After the chemical reaction, the graphene monolayer was transferred into deionized water using PET, to prevent it from highly doping due to the acidic solution. When the graphene sheet was transferred on the dielectric layer, it was necessary to take subsequent operations to fix the adsorbed graphene, such as heating the device at 140 °C for 30 min in ambient to evaporate excessive water. In the end, the PMMA was removed by acetone. Figure 2c illustrates the Raman spectrum of a piece of graphene transferred on 300 nm SiO_2_/Si. One can see that the D peak (≈1300 cm^−1^) indicates defects are extremely low, and the intensity ratio of G (≈1600 cm^−1^) to 2D peak (≈2700 cm^−1^) is 1:2, which satisfies the characteristics of SLG [35,36]. The inset is an optical micrograph of the polycrystalline graphene sheet. The dark spots dispersed on the surface represent the crystal nucleus. These experimental results imply that SLG was structurally complete and in good performance after transfer by the described method.

Finally, the ion-gel of 100 nm thickness, whose composition is referred to in the literature (Adamas Reagent Ltd., Shanghai, China) [37], is spin-coated on the whole specimen and solidified at 60 °C in an oxygen-free condition (KUANSONS Co., Shanghai, China) [20,38]. Before that, the source and drain electrodes were isolated from the ion-gel by pieces of polyimide tape to prevent any leakage from the gate. A Keithley 4200 semiconductor characterization system (Tektronix Inc., Beaverton, OR, USA) is utilized to investigate whether the FET configuration was working properly or not, and the additional conductive tape was attached to electrodes for convenient operation. It is illustrated in Figure 2d, that the current-voltage curve between the drain and source electrodes is almost linear when voltages sweep from −3 V to 3 V with a step of 0.2 V. At the same time, the current curve of the graphene channel inflecting at −3.5 V (Dirac point) was also shown in Figure 2d as a function of top-gate voltage (*V_TG_*) varying from −10 V to 6 V, while the drain voltage was fixed at 0.1 V. Therefore, the FET function of the device was confirmed to be effective. The charge neutrality point (CNP) shifts to a negative value, because the graphene flake with higher work function acts as an acceptor of electrons (n-dopants) when it contacts electrodes. According to the report [39], the work function ϕ of suspended graphene and the clean aluminum surface were 4.5 eV and 4.22 eV, respectively.

**Figure 3:**
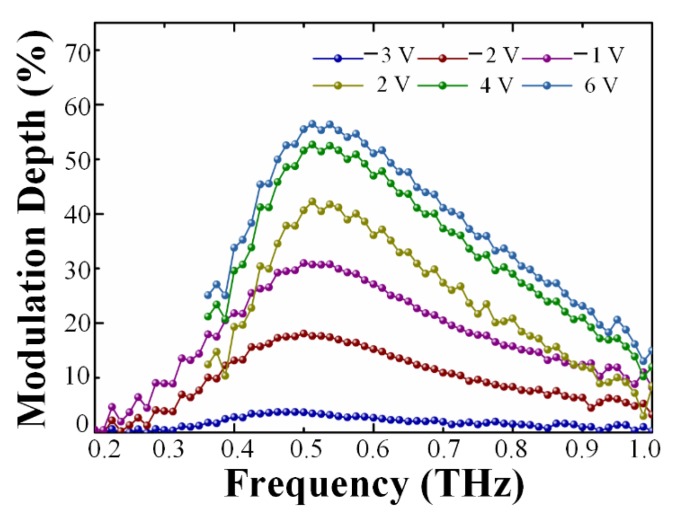
A series of modulation depth (*MD*) via different gate voltages of this device.

The modulation capability of this device was investigated with a THz-TDS system, from 0.2 to 1 THz [40]. The original experimental results were processed through Fourier transform, thus the analysis was mainly performed in the frequency domain. To evaluate the modulation performance of this novel device, *MD* is a crucial indicator, which is defined as:

(1) $$MD=(1\;-\;\frac{T_{Vg}}{T_{max}})\times 100\%,$$

where *T_V__g_* is the measured transmittance of a sample at a specific gate voltage, and *T_max_* is the maximum transmittance among different voltages. The experimental *MD* is demonstrated in Figure 3. The curves indicate that the optimum operating band is around 0.5 THz. Corresponding to the *T_max_* occurring at −3.5 V, the maximum MD is 55% at 6 V. The relationship between gate voltage and *MD* is non-linear. In detail, the increased rate of *MD* gradually slows down, with the applied voltages shifting away from the Dirac point.

## 3. Discussion

To analyze the device theoretically, we utilize the finite element method to establish a theoretical model of the device. Due to the periodic metamaterial in the functional region, the original model is simplified and represented by one cell with a periodic boundary in the horizontal plane. As for structure sizes, they all refer to the actual ones of the modulator, as shown in Figure 1c. The specific parameters not mentioned before are set as follows: the dielectric constant of PET $\varepsilon_{PET}=3$, the conductivity of the aluminum layer $\delta_{Al}=3.56\times 10^{7}\;S/m$, the dielectric constant of the aluminum oxide layer $\varepsilon_{Alumina}=6$ [41], and the dielectric constant of the solid electrolyte layer $\varepsilon_{Solid-electrolyte}=5.5$ [37]. The conductivity of graphene, $\sigma_{graphene}$, is defined by the formula [42]:

(2) $$\sigma_{graphene}\;\approx \;\sigma_{intra}=-i\frac{e^{2}K_{B}T}{\pi \hbar \left(\omega -i2\Gamma \right)}\left(\frac{E_{f}}{K_{B}T}+2\ln(e^{-\frac{E_{f}}{K_{B}T}+1})\right),$$

where *E_f_* is the chemical potential of the graphene, *e* = 1.602 × 10^−19^ C is the electron charge, *K_B_* is the Boltzmann’s constant, ℏ is the reduced Plank constant, ω is THz frequency, *T* = 300 K is the temperature, and Γ = 100 THz is the carrier scattering rate.

### 3.1. Solid Electrolyte Model

In order to analyze the device using a theoretical model, we need to figure out the relationship between the applied gate voltage and the corresponding Fermi level of SLG graphene. The electrolyte capacitance is the key cue to solving this problem.

**Figure 4:**
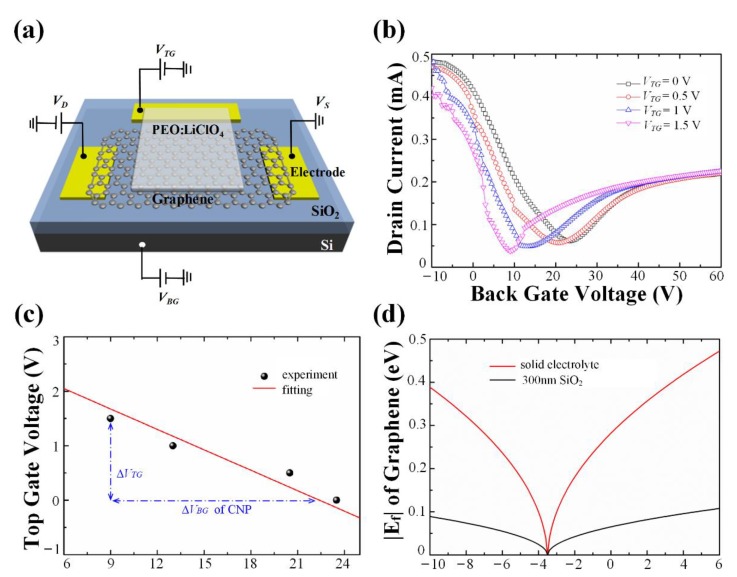
The configuration and experimental results revealing the relationship between graphene doping levels and gate voltages. (**a**) Schematic of a dual-grating-gate configuration with public source-drain electrodes. (**b**) Relationship between drain current and back-gate voltage (*V_BG_*) under different top-gate voltages (*V_TG_*). (**c**) Carrier-neutral points under various conditions. Experimental data is exhibited in black dots and then fitted with a red line. (**d**) Theoretical curves about the chemical potential (*E_f_*) of single-layer graphene doped by PEO:LiClO_4_ and 300 nm SiO_2_/Si respectively.

We adopted a so-called dual-grating-gate configuration, demonstrated in Figure 4a, to obtain the capacitance of PEO:LiClO_4_ [43]. As the doping level of SLG is simultaneously controlled by thetop-gate and back-gate, the shift of CNP should follow the relationship defined by $C_{g}/C_{SiO_{2}}\;=-\left(\Delta V_{BG,CNP}/\Delta V_{TG}\right),$ where *C_g_* and *C_SiO__2_* are the capacitance of the solid electrolyte and the 300 nm SiO_2_/Si (0.0121 μFcm^−2^) respectively [18,19,20], and *V_BG,CNP_* is the back-gate voltage of CNP. Figure 4b shows several transfer characteristic curves measured by dual-grating-gate configuration when the top-gate is set as 0 V, 0.5 V, 1 V, and 1.5 V, respectively. Accompanied by an increase in *V_TG_*, the Dirac point moves to left gradually. The specific movement rule of CNP is shown in Figure 4c. The value of the fitting curve, $-\left(\Delta V_{BG,CNP}/\Delta V_{TG}\right)$, is 10, which means the capacitance of spin-coated electrolyte is 10 times larger than that of a 300 nm SiO_2_ plane-parallel capacitor. Eventually, the free carrier concentration of electrolytes under various *V_TG_* could be theoretically predicted by the following equation [20,27,38].

(3) $$V_{g}-V_{CNP}=\frac{\hbar \left|\nu_{F}\right|\sqrt{\pi n}}{e}+\frac{ne}{C_{g}}\;,$$

where *V_g_* is gate voltage, *V_CNP_* is the CNP voltage, $\nu_{F}$ is Fermi velocity set as 1.1 × 10^6^ m/s [44], and *n* is the actual carrier density. Moreover, when Equation (3) combines with Equation (4) below, the *E_f_* could be calculated as a function of the gate voltage in Figure 4d.

(4) $$E_{f}=\hbar \left|\nu_{F}\right|\sqrt{\pi n}.$$

According to Figure 4d, by comparison to SiO_2_/Si, solid electrolyte requires lower voltage at the same doping level. Particularly, the *V_TG_* of −10 V, −4.5 V, 0 V, and 5 V corresponds to the chemical potential of 0.39 eV, 0.15 eV, 0.28 eV, and 0.45 eV, respectively.

### 3.2. Metamaterial Enhancement

In this section, we aim to prove that the proposed metallic structure can indeed promote modulator performance with single-layer graphene.

**Figure 5:**
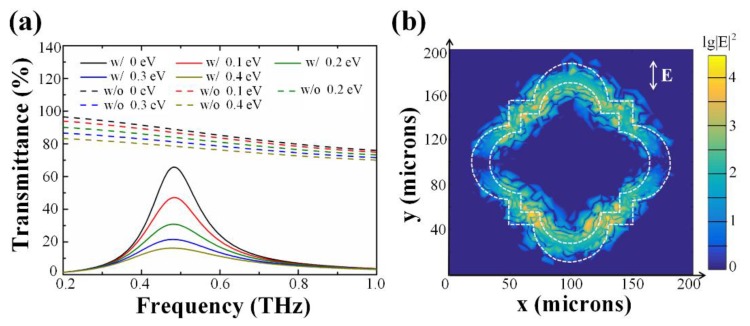
The differences in simulation results with and without the metamaterial layer. (**a**) Simulated THz transmittances with different Fermi levels. The configurations with the metallic layer are set as the normal group, and the rest are assigned as the reference group. In the legend, w/ and w/o are short for “with” and “without”. (**b**) Enhancement ratio of the electric field between the normal and the contrast group, both with unadulterated graphene at 0.48 THz.

In Figure 5a, three conclusions can be drawn from the simulated transmittances with and without metal structure. Firstly, a resonance mode of the metamaterial array was excited at 0.48 THz. Secondly, regardless of whether there was a metallic film or not, the transmittance generally decreased with the Fermi level increments. Thirdly, the theoretical calculation showed that the *MD* of the device with the metamaterial layer was deeper than the one without at resonance frequency. To be specific, in 0.48 THz, the *MD* rises from 11.6% to 75.4% within 0.4 eV after employing the metamaterial layer.

Moreover, the enhancement ratio of the electric field in the graphene layer was demonstrated in Figure 5b to explain the mechanism of how the flower-shaped structure contributes to performance improvement. The *E_f_* of graphene is 0 eV, and the frequency is 0.48 THz in the parameter settings. It was observed that the local field intensity near the metallic slits was enhanced, with an average of 10^2^ orders, compared with that of the control group. Further study showed that, accompanied by the gradual increasement in the Fermi level, the resonance coupling strength receded evidently.

In conclusion, the proposed metamaterial can effectively strengthen the local electric intensity around the SLG at the resonance frequency. Consequently, the dynamic modulation ability of SLG can be magnified by the enhanced field. Therefore, the flower-shaped structure benefits the deep modulation of THz transmittances by monolayer graphene.

### 3.3. Overall Analysis

**Figure 6:**
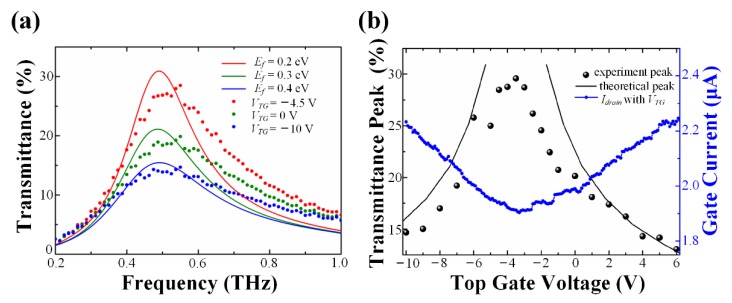
Theoretical results compared with experimental ones in the proposed modulator. (**a**) THz transmission spectra with different Fermi levels illustrated, both in practice and in theory. According to the previous analysis, the Fermi level is 0.15 eV, 0.28 eV, and 0.39 eV when *V_TG_* is set as −4.5 V, 0 V, and −10 V, respectively. (**b**) The transmittance summits of prediction and of measurement versus gate voltages, shown by black lines and dots, respectively. The transfer characteristic curve of the designed modulator was demonstrated at source-drain voltage 0.1 V, in the blue dots and line.

Experimental transmission spectra are compared with simulated ones with a higher level of *E_f_* in Figure 6a. One can see that the tendencies exhibited are consistent with each other. Furthermore, at similar *E_f_* of graphene, the theoretical transmittance is slightly higher than the measured ones, because of the non-loss material settings of the solid electrolyte and the dielectric layer in the analytical model. According to Figure 6a, the resonance peak frequencies are located at 0.5 THz; this is slightly different from the one calculated in Figure 5a. The shift is mainly attributed to the widening of the minimum etched groove (≈1 μm) during fabrication.

Additionally, the summits of transmittance versus voltages are illustrated in Figure 6b, and the transfer characteristic curve of graphene is also shown as a reference.

According to previous studies, the relationship between transmittance (*T*) and electric resistance of a graphene flake (*R*) could be described by Equations (5) and (6) for a FET-type modulator [25,45,46,47].

(5) $$T\propto \frac{W}{L}R,$$

(6) $$\frac{1}{R}=\frac{W}{L}C_{g}\mu \left|V_{g}-V_{Dirac}\right|+\frac{1}{R_{Dirac}},$$

where *C_g_* is the gate capacitance of FET, *μ* is the carrier mobility, *V_g_* is the gate voltage, *V_Dirac_* and *R_Dirac_* are the corresponding voltage and resistance of graphene in Dirac point, and *L* and *W* are the length and width of the graphene strip, respectively.

Combining the two equations above, we could conclude that the transmittance reaches the maximum at a gate voltage equal to *V_Dirac_*, which is in good agreement with Figure 6b [48]. Moreover, when *V_g_* applied shifts away from the *V_Dirac_*, the transmittance would gradually decrease.

The peak values of theoretical transmittances are extracted and drawn in black lines, while the experimental ones are shown in black dots. It was noted that the lines were only consistent with dots when the graphene Fermi level was higher than 0.17 eV. In other words, the experimental CNP no longer corresponds to 0 eV, and the Dirac point is no longer a single dot. As a consequence, the maximum transmittance of the experiment is only about 30% in Figure 6b, which is lower than the simulated value (66%) in Figure 5a. This difference is reasonable for large-area graphene films according to prior study [49]. The electron-hole puddles observed in large-scale graphene could evidently influence the residual carrier concentration around Dirac point [50] and lead to the absence of the conic point. Structural disorder, charged impurities, molecule doping, etc. will also increase the residual carrier density. A comparison of performance among this device and the previous is presented in Table 1, which shows the advantages of this work.

## 4. Conclusions

In summary, we propose a top-gated THz device to realize the high-performance modulation of single-layer graphene. In order to improve the poor performance induced by weak interaction and large applied voltages, this modulator employs a metallic metamaterial and solid electrolyte layer to target the flaws. Specifically, the enhanced electric field excited by the flower-shaped structure can strengthen the interaction when the terahertz wave goes through the single-layer graphene, and the solid electrolyte can effectively elevate the doping level of graphene at the same gate voltage. Due to these two optimizations, the modulator efficiency was notably increased. It was verified that the maximum modulation depth is ≈78.7% in theory and ≈53% in experiment within 5 V of gate voltages in this paper. Besides, the fabrication of the device is also inexpensive. The metamaterial layer in this work could be replaced by a customized one for a specific application in the future.

## Figures and Tables

**Table 1:** The performance comparison between different kinds of THz amplitude modulators. In this table, w/ and w/o are short forms of “with” and “without”.

THz Amplitude Modulator	Modulation Depth [%]	Operating Voltage Interval [V]
w/ metamaterial, w/o PEO:LiClO_4_ [51]	2; 22	−20 to 10
w/ metamaterial, w/o PEO:LiClO_4_ [13,17]	53; 50	0 to 16; −20 to 20
w/o metamaterial, w/ PEO:LiClO_4_ [48]	20	−3 to 3
w/ metamaterial, w/ PEO:LiClO_4_ (in this work)	53	−3.5 to 5
